# Living biointerfaces based on non-pathogenic bacteria support stem cell differentiation

**DOI:** 10.1038/srep21809

**Published:** 2016-02-23

**Authors:** Jake J. Hay, Aleixandre Rodrigo-Navarro, Karoliina Hassi, Vladimira Moulisova, Matthew J. Dalby, Manuel Salmeron-Sanchez

**Affiliations:** 1 Division of Biomedical Engineering, School of Engineering, University of Glasgow, UK; 2 Centre for Cell Engineering, Institute of Molecular, Cell and Systems Biology, University of Glasgow, UK

## Abstract

*Lactococcus lactis*, a non-pathogenic bacteria, has been genetically engineered to express the III_7–10_ fragment of human fibronectin as a membrane protein. The engineered *L. lactis* is able to develop biofilms on different surfaces (such as glass and synthetic polymers) and serves as a long-term substrate for mammalian cell culture, specifically human mesenchymal stem cells (hMSC). This system constitutes a living interface between biomaterials and stem cells. The engineered biofilms remain stable and viable for up to 28 days while the expressed fibronectin fragment induces hMSC adhesion. We have optimised conditions to allow long-term mammalian cell culture, and found that the biofilm is functionally equivalent to a fibronectin-coated surface in terms of osteoblastic differentiation using bone morphogenetic protein 2 (BMP-2) added to the medium. This living bacteria interface holds promise as a dynamic substrate for stem cell differentiation that can be further engineered to express other biochemical cues to control hMSC differentiation.

The extracellular matrix (ECM) is a complex array of polysaccharides, proteins (such as fibronectin, laminins, collagen, vitronectin) and growth factors (GF) that provide mechanical and biochemical support to cells, and plays a critical role in cell fate determination^1^,^2^,^3^. Cell-ECM interaction takes place through membrane-bound proteins such as integrins and growth factor receptors^4^. Fibronectin and GF receptors are involved in cell dynamics and sensing the environment, translating extracellular events into cytoplasmic activation of different signalling pathways^5^. Such interactions modulate a variety of cell responses that include adhesion, proliferation, migration and ultimately survival and differentiation^4^,^5^.

Our aim is to exploit the extracellular matrix/cell receptors interaction in the design of materials of biomedical interest. This interaction takes place through an intermediate layer of proteins such as fibronectin^6^,^7^, vitronectin^8^,^9^, laminin^10^,^11^ collagens^12^,^13^ or synthetic peptides adsorbed on synthetic surfaces used for *in vitro* cell culture. However, due to the inherent static properties of surface functionalization through either protein adsorption or covalent protein binding on surfaces, attempting to recreate the dynamic nature of the ECM has become a major research driver. Some authors propose the use of materials with the ability to modify its physical^14^,^15^,^16^ or chemical^17^,^18^,^19^,^20^,^21^,^22^ properties under external stimuli to mimic, to a certain degree, the dynamic properties of the ECM.

Actual applications of this strategy display the adhesive peptide RGD through several approaches, such as protease-cleavable moieties that expose the peptide^17^, surfaces where the RGD is selectively exposed via reversible attachment of leucine zippers^23^ or where RGD is exposed when light with the appropriate wavelength cleaves a blocking moiety and renders it accessible to integrins^24^,^25^.

None of the current strategies can be considered a true, interactive biointerface where cell fate is controlled by signals released in a spatiotemporal manner. Ideally, these interfaces should also be able to enable crosstalk with mammalian cells establishing a series of feedback loops aimed at directing cell behaviour. In this report, our hypothesis is that non-pathogenic bacteria can be engineered to play such a role. In previous work^26^, we showed a system where *Lactococcus lactis* subsp. *cremoris*, a gram-positive, non-pathogenic bacteria was genetically engineered to express the III_7–10_ fragment of the human fibronectin (FN III_7–10_ - from here denoted as *L. lactis*-FN) in its cell wall. This III_7–10_ fragment itself induces cell behaviour such as adhesion, survival and proliferation and differentiation in several cell types, including mesenchymal stem cells.

This work investigates the potential of this living biointerface to support and control stem cell adhesion, survival and differentiation in long-term cultures. The novelty of this system is that it is able to support mammalian cell cultures for up to 4 weeks, and that it can be modified to express any desired biochemical cue as growth factors or small molecules which can be utilised to induce cell differentiation.

Human mesenchymal stem cells (hMSCs) from the bone marrow are multipotent cells that can, under adequate conditions, differentiate into osteogenic, chondrogenic, adipose and reticular lineages^27^. hMSC differentiation can be triggered by many factors, including material properties such as stiffness^28^,^29^, nanotopography^30^ and biochemical cues^31^,^27^. We use BMP-2, a member of the bone morphogenetic protein family, to help regulate the hMSC phenotype. It is a potent cytokine that mediates cell proliferation and differentiation of hMSCs, and has been shown to play an essential role in bone morphogenesis^32^, activating either runt-related transcription factor 2 (RunX2) and/or Osterix activation, mediated by Smad proteins^33^,^34^,^35^. The cell-wall displayed FNIII_7–10_ in *L. lactis* and the use of exogenous BMP-2 allows long-term maintenance and functionality of both cell populations (bacteria and MSCs) and osteogenesis when required. The challenge is to control the simultaneous and stable culture of bacterial and stem cells. Moreover, *Lactococcus lactis* lacks lipopolysaccharide production^36^ that could interfere with the mammalian cell signalling routes and enables the direct interaction of the membrane bound proteins and the mammalian integrins. This lack of LPS production has been exploited in the production of recombinant proteins in *L. lactis* with a greater purity and lack of endotoxins when compared to *E. coli*.

## Results

**Figure 1:**
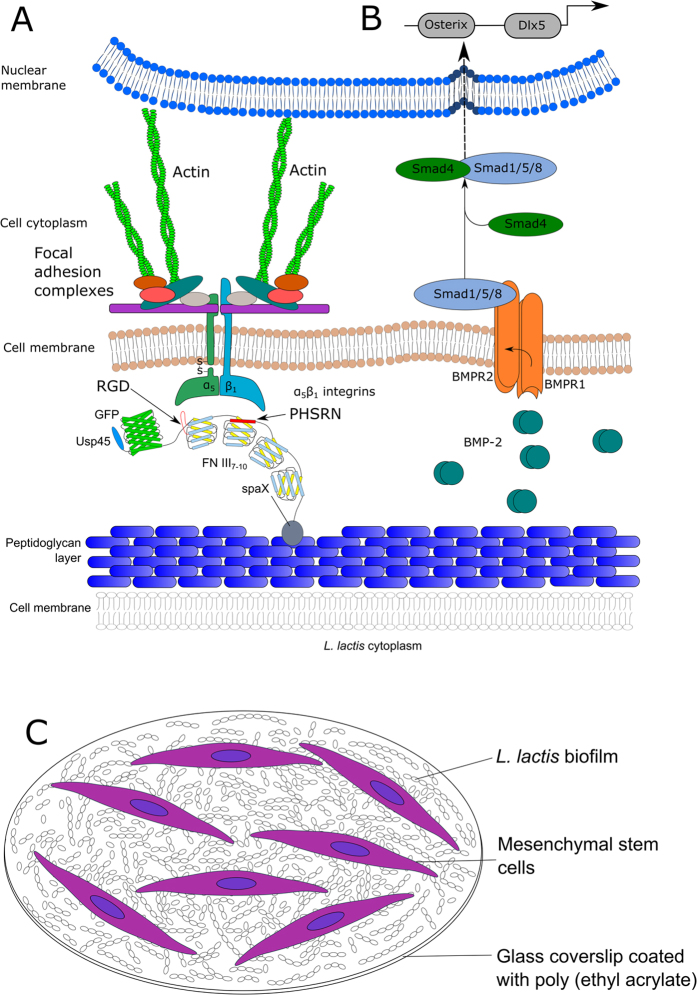
Conceptual overview of the system. (**A**) *L. lactis* biofilm expressing the III_7–10_ fragment of the human fibronectin on its cell wall, fused to green fluorescent protein (GFP) as a reporter, acting as a biointerface for bone marrow-derived human mesenchymal stem cells. (**B**) Recombinant human BMP-2 (rhBMP-2) was added in the cell culture medium at 100 ng/mL to induce osteogenic differentiation. BMPR1/2 signalling through the Smad pathway leads to activation of the transcription factors Osterix, RunX2 and Dlx5 which induces expression of proteins involved in osteogenic differentiation. (**C**) *L. lactis* biofilm is able to support hMSCs growth and differentiation for up to 28 days, while keeping good viability values and stable morphology.

Figure 1 shows the conceptual scheme of this work. A *L. lactis* biofilm expressing the III_7–10_ fragment of the human fibronectin on its cell wall, fused to green fluorescent protein (GFP) as a reporter protein is used as a substrate for cell culture. Recombinant human BMP-2 (rhBMP-2) is added to the cell culture medium at 100 ng/mL to induce osteogenic differentiation. FNIII_7–10_ contains the arginine-glycine-aspartic acid (RGD) motif in the III_10_ repeat and the PHSRN or synergy sequence in the III_9_ repeat. These two motifs have been shown^37^ to interact with the α_5_β_1_ integrin in a specific fashion, favouring osteogenic differentiation in human MSCs^38^. It has been shown by Moursi *et al.* that the binding of α_5_β_1_ to FN is essential for osteoblast-specific gene expression in osteoblast cell cultures^39^. In contrast, the α_v_β_3_ integrin has been shown to down-regulate osteoblastic differentiation and matrix mineralisation^40^. This highlights that the α_5_β_1_ integrin is a likely candidate to transduce at least some of the regulatory signals required for osteogenesis. Additional signals are nonetheless required to induce osteogenesis, such as the addition of growth factors in the culture medium, such as BMP-2. Martino *et al.* have shown that differentiation of MSCs is greatly enhanced when BMP-2 and the α_5_β_1_ integrin are stimulated synergistically when compared with only growth factor^41^. The addition of FNIII_7_ and FNIII_8_ was chosen as there are several literature references that indicate that the whole III_7–10_ fragment is biologically active^42^,^43^. From our point of view it gives a higher degree of flexibility to the active III_9_ and III_10_ sequences of FN and it moves these active sites away from the bacterial membrane.

Bacterial viability was assessed using different antibiotics over four weeks, the maximum time period we used to differentiate hMSCs.Then, the interaction of hMSCs with the biofilm was explored through scanning electron microscopy (SEM) and vinculin immunostaining followed by observation of terminal differentiation to osteoblasts. To study osteogenic differentiation, osteocalcin expression and ECM mineralisation through phosphate deposition were studied.

### Bacterial viability

**Figure 2:**
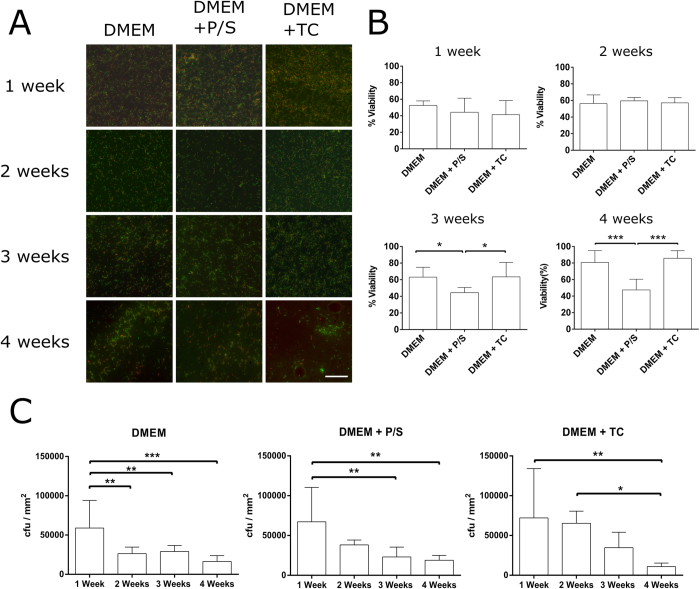
*L. lactis* biofilm viability. Biofilms of *L. lactis*-FN were produced on a poly (ethyl acrylate) (PEA) surface and cultured for 1 to 4 weeks with DMEM, DMEM supplemented with 100 U/mL of penicillin-streptomycin (DMEM+P/S) and DMEM supplemented with 10 μg/mL tetracycline (DMEM+TC). (**A**) After the selected time points, biofilms were washed and their viability assessed using the commercial BacLight viability kit (Life Technologies). This kit stains viable cells in green and non-viable cells in red. Viability was calculated by analysing the total amount of cells stained in green versus the amount of cells stained in red and green. (**B**) After 3 weeks, viability values were found to be higher in biofilms cultured with DMEM or DMEM+TC than in biofilms cultured with DMEM+P/S. This result might be attributed to the fact that P/S is bacteriolytic in comparison to TC, which is bacteriostatic. There is an increase in viability values of the 4 week biofilms that can be attributed to the detachment of non-viable cells. (**C**) Biofilm coverage was calculated as the percentage of the surface covered by bacteria, expressed as cfu (colony forming units) per square milimeter. We found that a large proportion of the biofilm had detached as the culture time increased, explaining the rise in viability found in B. This results suggests that biofilms produced on PEA, a hydrophobic polymer, can serve as a substrate for long-term stem cell culture. Scale bar size is 50 μm. Data is presented as mean ± SD, and was analysed with a one-way ANOVA test with a Tukey post-hoc test. Statistical significance levels are *p < 0.05 and ***p < 0.001.

It was important to deduce the viability of *L. lactis-*FN after 4 weeks of growth as the stability of the living interface on the material surface modulates cell adhesion. Figure 2 shows bacterial viability after 1, 2, 3 and 4 weeks. Due to the nature of the bacteria-surface interaction, governed by non-specific Van der Waals and Lewis acid-base forces, the choice of an appropriate surface is of critical importance^44^. In our previous work, *L. lactis* biofilms were developed on glass, a hydrophilic surface where, although biofilms develop well while keeping bacterial viability values over 60%, its finite life cycle and relatively weak adhesion forces were not enough to support long-term cell cultures. After 4 days, a significant part of the adhered cells started to detach^45^.

To overcome this obstacle, poly (ethyl acrylate) (PEA), a polymer previously used as mammalian cell culture substrate^46^ was found to be particularly well suited for bacterial adhesion^44^,^47^ allowing the development of biofilms which have been shown to last the 28 days required for osteogenic hMSC differentiation (Fig. 2). Nevertheless, *L. lactis* proliferate continuously if nutrients are available to metabolise and convert any carbon source present into lactic acid^48^. The build-up of this organic acid unavoidably leads to a decrease in the pH of the culture medium, which is detrimental for mammalian cell viability.

**Figure 3:**
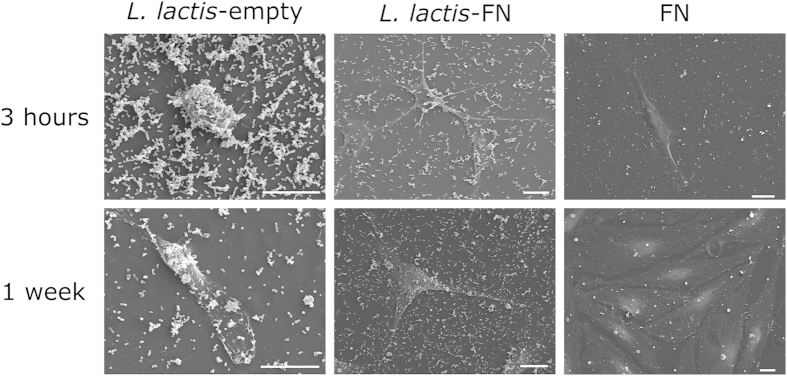
Morphological study of the hMSCs by SEM. hMSC behaviour on the *L. lactis*-FN and *L. lactis*-empty biofilms, and FN-coated surface was assessed by scanning electron microscopy. Cells were cultured for 3h in absence of FBS and for one week with 1% FBS, in this case to ensure cell viability. hMSCs cultured for 3h on the *L. lactis*-empty biofilm kept a round shape, while in the *L. lactis*-FN and in the FN-coated surface showed adherence and spreading. On the other hand, in the 1 week cultures, due to the need to use FBS to ensure viability, cells showed an elongated shape with evidences of adhesion behaviour. Nevertheless, hMSCs showed different features in the *L. lactis*-FN compared to the *L. lactis*-empty biofilm, this behaviour can be attributed to the presence of the III_7–10_ fragment exposed on the *L. lactis* cell wall. Cells cultured on the FN-coated surface displayed higher proliferation ratios. Scale bar size is 20 μm.

We have previously outlined that tetracycline (TC) used at 10 μg/mL is sufficient to slow down bacterial metabolism without impacting mammalian cell behaviour up to 4 days^45^. TC inhibits bacterial protein synthesis by binding to the 30S subunit of bacterial ribosomes blocking the attachment of the charged aminoacyl tRNA to the A-site^49^. Due to the differentiation characteristics of hMSCs it was necessary to elongate this time course up to 4 weeks. To increase efficiency, a mixture of penicillin/streptomycin (P/S) at 100 U/mL was also tested. P/S acts by interfering with the synthesis of the peptidoglycan cell wall and leads to cell lysis^50^ which is detrimental to biofilm viability. We only tested *L. lactis-*FN and not *L. lactis* not expressing any FN (here on denoted *L. lactis-*empty) as previously published work^45^ showed that the two strains showed markedly similar results. Further to this, *L. lactis*-empty induces no cell adhesion (as shown in Fig. 3) and therefore, no further cellular processes can occur on this strain.

These approaches were tested to prevent initial bacterial cell death with the aim to improve the preservation of the biofilm in a bacteriostatic manner. *L. lactis* could be seen to have formed stable biofilms at all-time points on the material surface (Fig. 2). Bacterial viability was constant at approximately 50% after 1 week with the highest viability found in the antibiotic free samples. After 2 weeks, viability was again seen to be similar between the samples, and increased to approximately 55%. After 3 weeks the viability for DMEM + P/S (44%) was found to be significantly different from DMEM (63%) and the DMEM + TC (63%). This difference was further confirmed by the 4 week results, again, the viability for DMEM + P/S (47%) was significantly lower than both the DMEM (81%) and DMEM + TC (85%) samples (Fig. 2B).

The data suggests that viability increases as a function of time. However, upon quantifying the surface coverage of the biofilm in colony forming units (Fig. 2C), it became apparent that a portion of the biofilm had detached from the substrate. Non-viable cells would detach from the surface and the presence of antibiotic would prevent bacterial proliferation, thus increasing the ratio of viable to non-viable bacteria. The bacterial biofilm was found to be present after 4 weeks of culture (Fig. 2), with a similar bacterial morphology to shorter time points. It can be inferred from this that FN is still present on the bacterial membrane allowing MSC adhesion.

### hMSC adhesion and morphology

Cell adhesion and morphology was assessed through SEM and focal adhesion immunostaining against vinculin. hMSC ability to interact with *L. lactis*-FN compared to FN coated surface and *L. lactis*-empty were first assessed. hMSC spreading after 3 hours was found to be directly related to the availability of FN to the hMSCs. Figure 3 shows SEM images after 3 hours and 1 week of hMSC culture. From these images, it became apparent that the FN fragment in the bacterial membrane is essential to develop adhesion structures after 3 hours, since hMSCs cultured on top of *L. lactis*-empty biofilms kept a rounded shape, with no sign of adhesion. The morphology of the hMSCs on the FN coated surface and *L. lactis-*FN appear similar. It is clear that the initial cell interaction determines cell morphology and it is distinctly affected by the availability of the FN fragment on the bacterial membrane. After 1 week, cell morphology on all surfaces becomes more similar. This is likely due to the addition of 1% foetal bovine serum (FBS) to the culture media, which is necessary to ensure the viability of the hMSCs in the long-term.

**Figure 4:**
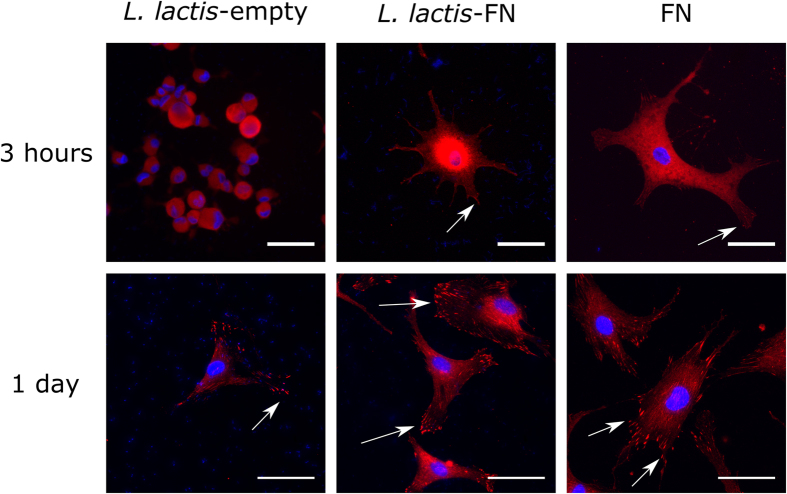
Adhesion assessment of hMSCs cultured on *L. lactis*-empty, *L. lactis*-FN and FN-coated surface. Cells were cultured for 3 hours in absence of FBS and 1 day with 1% FBS with an initial density of 5,000 cells/cm^2^. After selected times, cells were fixed and immunostained against vinculin (red) and DAPI (blue). hMSCs cultured for 3 hours in the *L. lactis*-empty biofilm showed no sign of adhesion, keeping a round shape, while in the *L. lactis*-FN and FN surfaces there is evidence of spreading and adhesion, although focal adhesion complexes are not fully developed. After 1 day, there are focal adhesion (FA) complexes in the three different conditions; the presence of FA in cells cultured on *L. lactis*-empty biofilm is most probably due to the presence of FBS in the medium. Cells cultured on the *L. lactis*-FN biofilm and FN-coated surface displayed more developed FA complexes compared to *L. lactis*-empty biofilm, suggesting that the already present fibronectin in the surface (either on the *L. lactis* cell wall or grafted on the surface) enhances the development of this FA complexes. Scale bar size is 50 μm.

After initial SEM morphology studies (Fig. 3), hMSCs were stained for vinculin, a protein present in focal adhesion complexes, to explore any changes in focal adhesion morphology across the surfaces. Figure 4 shows vinculin staining on the surfaces after 3 hours and 1 day. After 3 hours, hMSCs cultured on *L. lactis-*empty display a rounded morphology, with no signs of adhesion whereas hMSCs cultured on the FN coated surface and *L. lactis-*FN display evidence of spreading and adhesion; the nascent focal adhesions could be seen at the edges of the cell lamellae (see arrows in Fig. 4). After 1 day, cells on all surfaces have spread and adhered to the surface, as shown by positive vinculin staining in the cell lamellae. The presence of vinculin in the *L. lactis-*empty sample is likely due to the addition of FBS to the media. FBS contains many proteins essential for long term cell culture and also comprises many cell adhesive proteins which become adsorbed to the surface, allowing cell adhesion.

However, critically, hMSCs cultured on *L. lactis*-FN and the FN coated surface displayed far more developed focal adhesions, showed by the dashed morphology compared to *L. lactis*-empty where adhesions were predominantly punctate, dot shaped focal complexes^51^. These differences in the organisation of focal adhesions cancel out after 3 days of culture (Supplementary Fig. S2).

A time lapse experiment was set up to record hMSC behaviour over the different surfaces shown in videos 1 and 2 (supplementary material). hMSCs on the *L. lactis-*FN biofilm were able to spread, migrate and proliferate 30 minutes after seeding. However, cells on the *L. lactis-*empty were seen to stay in a rounded morphology and drift randomly across the surface for the first two hours of culture before adhering. The presence of the FN fragment allows a faster MSC adhesion and spreading, resulting in higher mobility of cells on top of the *L. lactis*-FN biofilm when compared against cells cultured on top of *L. lactis*-empty biofilm supplemented with 1% FBS.

### Osteogenic differentiation

**Figure 5:**
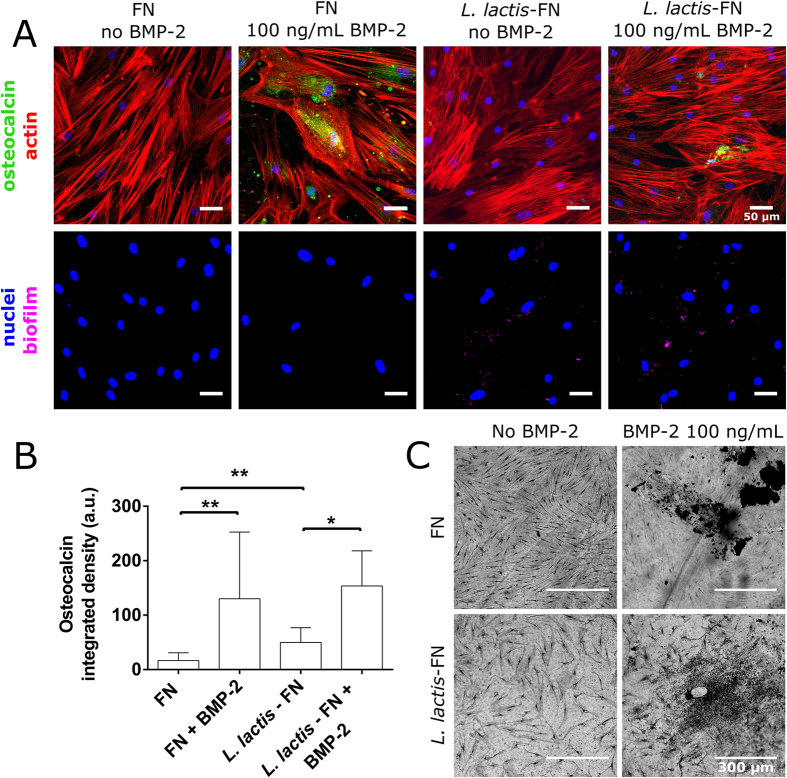
Osteogenic differentiation assessment through osteocalcin and von Kossa stainings. (**A**) hMSCs were immunostained for osteocalcin (green) and actin (red) (top row). In the bottom row, reconstructed DAPI images for cell nuclei (blue) and bacterial DNA (purple). From left to right, cells were cultured on a FN-coated surface with and without BMP-2 at 100 ng/mL and on *L. lactis*-FN biofilm, again with and without BMP-2 at 100 ng/mL. Total integrated density corresponding to the green channel (osteocalcin) was quantified using ImageJ. Scale bar is 50 μm. (**B**) Graph shows OCN area per cell with standard deviation of the sample sets, N ≥ 250 cells were analysed for each condition. Integrated density corresponding to osteocalcin was found to be significantly higher in the samples treated with BMP-2 than in the samples without BMP-2. Data is presented as mean ± SD and analysed with ANOVA with a Tukey post-hoc test. Significance levels are *p < 0.05 and **p < 0.01. (**C**) Mineralisation (phosphate deposition) was assessed with a von Kossa staining. hMSCs were cultured for 28 days on a surface coated with FN (top row) and on *L. lactis*-FN biofilms (bottom row). After staining, cells were imaged in a Zeiss AxioObserver Z1 microscope using phase contrast. Cultures treated with BMP-2 showed black areas corresponding to phosphate deposits produced by the hMSCs, while in the untreated cultures there is no evidence of mineralization. Scale bar size is 300 μm.

BMP-2 was next used to ascertain the ability of hMSCs to be differentiated towards an osteogenic phenotype on the *L. lactis-*FN living interface. The bone specific ECM protein osteocalcin (OCN) as well as phosphate deposition (von Kossa) as part of calcium phosphate bone mineralisation were used to determine hMSC reaction to BMP-2. After 21 days, hMSCs were immunostained for OCN. Cultures exhibited similar cell density on all samples but showed negligible OCN production in samples not treated with BMP-2. Conversely, samples treated with BMP-2 displayed much higher OCN staining intensity by a factor of 7.6 and 3 on the FN coated surface and *L. lactis*-FN surfaces respectively (Fig. 5A,B).

In Fig. 5A, bottom row, the biofilm is still visible and marked as purple dots together with the cell nuclei, in blue. It is obvious that the bacterial density is lower than at the beginning, where the coverage of the biofilm can be as high as 27%, but in this case in particular, where *L. lactis* is providing only the adhesion cue, the importance of the biofilm is less essential than the differentiation cues provided by BMP-2 supplemented in the medium. Moreover, sample processing associated to the immunofluorescence protocol wash out a majority of the attached biofilm.

A second population of hMSCs were grown for 28 days before analysing phosphate deposition. Osteoblasts deposit phosphate as a primary step of bone development and therefore this process is suggestive of terminal osteogenic differentiation. As can be seen from Fig. 5C, phosphate deposition is distinctly higher (black deposits) in the samples treated with BMP-2 when compared to the control samples.

Together, the results confirm the ability of the living interface to support long-term MSC viability and trigger functionality, including mature differentiation with deposition of mineralised matrix.

## Discussion

Engineering the cellular microenvironment to direct stem cell behaviour is of fundamental importance for biomedical applications aimed at mimicking cell behaviour in its natural niche. It is known that cells interact with their environment, the ECM, through integrins^4^ and growth factor receptors resulting in biochemical signaling cascades through various signal transduction molecules including focal adhesion kinase (FAK)^38^. Collectively, these different signals dictate a plethora of responses already hardwired into the cells genes related to adhesion, migration, survival, proliferation and differentiation, in a dynamic and highly regulated fashion.

In this report, we propose a system in which mammalian cells can grow on engineered non-pathogenic bacteria, and that this population of modified bacteria can sustain and direct hMSC differentiation (e.g. towards an osteoblastic fate). This genetically engineered strain of *L. lactis* expresses the III_7–10_ fragment of human fibronectin tethered to its peptidoglycan layer by the *Staphylococcus aureus* protein A, making it available for mammalian cells integrin binding.

This fragment contains the RGD adhesion and the PHSRN synergy motifs. RGD interacts with a wide variety of integrins but, when combined with the synergy motif, a specific interaction with α_5_β_1_ takes place. Signalling initiated by this specific integrin has been linked to enhanced osteogenic differentiation in hMSCs^52^. Besides FNIII_7–10_ induced signalling, we have used BMP-2, a cytokine member of the bone morphogenetic protein family that is known to be a potent osteogenic inducer. The combination of FNIII_7–10_ expressed in the bacterial cell wall and BMP-2 added to the culture medium has proven effective in inducing osteoblastic differentiation in hMSCs, assessed by immunostaining against the bone specific protein osteocalcin and phosphate deposits, measured with von Kossa staining. Since hMSC differentiation needs 21 to 28 days, it is imperative to develop the *L. lactis* biofilm to support this extended culture time. *L. lactis* biofilms were first developed on glass, a hydrophilic surface, and were found to last at least 4 days in co-culture with mammalian cells^27^. For a successful co-culture we needed a stronger interaction between the biofilm and the underlying surface. In this case, surface hydrophobicity plays a role, as shown by the extended Derjaguin-Landau-Verwey-Overbeek model (XDLVO)^53^. We switched to a more hydrophobic surface, poly (ethyl acrylate) thin films coated on glass substrates.

Poly (ethyl acrylate) is a hydrophobic, non-biodegradable synthetic polymer that showed excellent adhesive properties for *L. lactis*. Surfaces coated with this polymer supported good bacterial viability, in the range of 50–60%, similar to biofilms developed on glass. Biofilms developed on PEA^46^ are able to remain stable for at least 28 days, facilitated by the use of the bacteriostatic antibiotic tetracycline (TC). TC is used to impede bacterial metabolism and prevent medium acidification via lactic acid production, which results in increased longevity of the biofilm.

The rationale of using poly (ethyl acrylate) instead of glass is due to the nature of the interaction between the bacterial cell wall and the underlying surface. Our *L. lactis* strain MG1363 is a derivative of *L. lactis* subsp. *cremoris* TIL672, which features a hydrophobic surface^54^. It is known that fibronectin strongly adheres to hydrophobic surfaces; the presence of a fibronectin fragment in the cell wall surface might be increasing its hydrophobicity. We also observed that this strain, when grown in M17, tends to co-aggregate, which is an indicative of its hydrophobic nature. The XDLVO model^53^ explains the interaction in terms of surface free energy of the interacting surfaces, and considers the solid-bacteria, solid-liquid and bacteria-liquid interfaces when calculating the free energy. In this model, the interaction is not distance-dependent and an obvious conclusion is that interaction of hydrophobic surfaces are favoured over hydrophobic-hydrophilic surfaces. A feasible explanation at a molecular level is that interaction between two apolar moieties immersed in water is the consequence of the hydrogen-bonding energy of cohesion of the water molecules surrounding them, so at the end, the use of a hydrophobic surface is better for biofilm development, at least with this strain. In the future, for other strains, an evaluation of their hydrophobicity should be made to improve the biofilm development.

The combination of these factors, that is to say, improved biofilm stability, bacterial cell-wall exposed FNIII_7–10_ and its integrin-related signalling and the related BMP-2 induced signalling lead to improved osteogenic differentiation in hMSCs when compared to FNIII_7–10_ signalling alone. Differentiation was assessed in 21 and 28-day cultures through osteocalcin immunostaining and phosphate deposition (mineralization). The influence of the FNIII_7–10_ on hMSCs was also assessed by studying hMSC adhesion at various time points (between 3 hours and one week) by vinculin immunostaining and cell morphology studies with SEM.

This work has shown that engineered bacteria can sustain the long term growth and differentiation of stem cells as a fundamental step before developing the full potential of the system as a dynamic interface. We have shown that there is very little difference between a fibronectin coated surface and our modified bacterial surface in terms of cell adhesion and differentiation. This bacterial surface holds an advantage over a simple fibronectin coat, in that it can be further modified to produce a superfluity of proteins or growth factors that can be used to direct cell fate. Further genetic engineering on this strain is currently being undertaken to express, on demand, growth factors and achieve a controlled release of these growth factors in combination with other biochemical signals to control stem cell fate. This is advantageous over current static strategies in that the bacteria can be controlled to express proteins in a spatiotemporal manner; something that is currently unavailable in the literature. We propose a dynamic system that can not only direct cell adhesion, but will also be able to control cell differentiation.

## Methods

### Bacterial viability

To study viability of the bacteria, the BacLight LIVE/DEAD kit (Life Technologies) was used. Biofilms were produced using a previously described protocol^45^ and transferred to DMEM, DMEM with 100 U/mL P/S and DMEM with 10μg/mL TC. Biofilm viability was tested after 1, 2, 3 and 4 weeks. The biofilm was washed once with sterile NaCl 0.85% w/v solution and incubated for 30 minutes using SYTO9 (5 μM) and propidium iodide (30 μM) in NaCl 0.85% w/v. After this, the samples were washed with PBS and mounted using Vectashield without DAPI (Vector Laboratories, UK) and imaged immediately. The viability was determined as the ratio between the viable and total number of bacteria. Images were analysed with Fiji – ImageJ software.

### Human MSC Cell culture

Human bone-marrow derived MSCs (Promocell) were maintained in DMEM supplemented with 4.5 g/L glucose, 100 μM sodium pyruvate, 1 mM L-glutamine, 10% foetal bovine serum (FBS) and 100 U/mL P/S. After seeding, media was changed to DMEM supplemented with 1% or no FBS and 10 μg /mL tetracycline to inhibit bacterial metabolism and prevent culture medium acidification. Cultures were kept at 37 °C and 5% CO_2_ in a humidified atmosphere. Media was changed every 2/3 days. To induce differentiation, 100 ng/mL BMP-2 (R&D Systems, UK) was supplemented to the media along with every media change.

### Scanning Electron Microscopy

MSCs were seeded at 5,000 cells/cm^2^. After designated culture times, samples were fixed in 2% glutaraldehyde/2% paraformaldehyde/0.15M sodium cacodylate buffer/0.15% Alcian blue for 2 hours at 4 °C. After fixation, samples were washed with 0.15 M sodium cacodylate buffer 5 times and incubated for 1 hour in 1% osmium tetroxide/0.1 M sodium cacodylate buffer. Samples were then washed 3× in distilled water and stained with 0.5% uranyl acetate in distilled water for 1 hour, protected from light. Samples were washed with distilled water before dehydration through an ethanol gradient (30%, 50%, 70% and 90% for 10 minutes each) with four 5 minute washes in 100% ethanol to fully dry the sample. Samples were then loaded onto a POLARON E3000 Critical Point Dryer (supercritical CO_2_) for 80 minutes and sputter-coated with Au/Pd using a POLARON SC515 SEM COATER. Samples were imaged in a JEOL 6400 SEM with a gun voltage acceleration of 10 kV.

### Immunofluorescence

After desired time points cells were fixed using 4% formaldehyde in phosphate-buffered saline (PBS) at 37 ^o^C for 15 minutes. Fixative was removed and washed with PBS before the addition of permeabilising buffer (10.3 g of sucrose, 0.292 g of NaCl, 0.06 g of MgCl_2_, 0.476 g of HEPES buffer in 100 ml of water, pH 7.2, with 0.5 mL Triton X-100) for 5 minutes at 4 °C. Samples were then washed and blocked with 1% BSA (bovine serum albumin) in PBS for 1 hour at room temperature. This was followed by the addition of the primary anti-osteocalcin antibody (Santa Cruz Biotechnology, UK) diluted 1:50 in BSA 1% in PBS or monoclonal anti-vinculin antibody diluted 1:400 in PBS/BSA (Sigma, UK) at 37 °C for 1.5 hours. The samples were next washed with 0.5% Tween 20 in 100ml PBS (3 × 5 minute washes).

For osteocalcin, a secondary IgG monoclonal, horse-produced, biotinylated antibody (Vector Laboratories, UK) diluted 1:50 in PBS/BSA 1% was added for 1 hour (37 °C). Rhodamine-conjugated phalloidin (Life Technologies, UK) was added for the duration of this incubation diluted 1:50 in BSA 1% in PBS. Samples were then incubated with FITC-conjugated streptavidin diluted 1:50 in PBS/BSA 1% at 4 °C for 30 min, and given a final wash. For vinculin, a Cy3-conjugated rabbit anti-mouse secondary antibody (Jackson Immunoresearch) diluted 1:100 in PBS/BSA 1% was added for 1 hour, simultaneously with BODIPY FL phallacidin (1:100, PBS/BSA, Invitrogen, UK). Samples were mounted with Vectashield with DAPI (Vector Laboratories, UK) and imaged in a fluorescence microscope (Zeiss Axio Observer Z1).

### Von Kossa staining

hMSCS were seeded at 5,000 cells/cm^2^ and cultured on freshly prepared *L. lactis*-FN biofilms for 28 days. Cells were fixed with 4% formaldehyde in ultrapure water for 5 minutes and then a 5% silver nitrate solution in H_2_O was added to ensure the coverslips were fully submerged and exposed to UV light for 30 minutes. After washing in deionised water, 5% sodium thiosulphate was added to the samples for 10 mins and then samples were washed with warm tap water for 10 minutes. After another wash with deionised water, the samples were counterstained with nuclear fast red for 10 minutes and washed again with deionised water. Finally the samples were rinsed with 70% ethanol and observed in a phase-contrast optical microscope (Zeiss Axio Observer Z1).

## Additional Information

**How to cite this article**: Hay, J. J. *et al.* Living biointerfaces based on non-pathogenic bacteria support stem cell differentiation. *Sci. Rep.* **6**, 21809; doi: 10.1038/srep21809 (2016).

## Supplementary Material

Supplementary Information

Supplementary video 1

Supplementary video 2
