# Supplementary material for: Living biointerfaces based on non-pathogenic bacteria support stem cell differentiation
Source: Sci Rep. 2016 Feb 23;6:21809. doi: 10.1038/srep21809 (PMC4763179; doi:10.1038/srep21809)
Supplement: Supplementary Information [file srep21809-s1.pdf]

Living biointerfaces based on non-pathogenic bacteria support stem cell differentiation

Jake J. Hay[1,§], Aleixandre Rodrigo-Navarro[1,§], Karoliina Hassi[1], Vladimira Moulisova[1], Matthew J. Dalby[2] and Manuel Salmeron-Sanchez[1]*

[1] Division of Biomedical Engineering, School of Engineering, University of Glasgow, UK

[2] Centre for Cell Engineering, Institute of Molecular, Cell and Systems Biology, University of Glasgow, UK

* Corresponding author, Manuel.Salmeron-Sanchez@glasgow.ac.uk

§ These authors contributed equally to this work.

Supplementary material

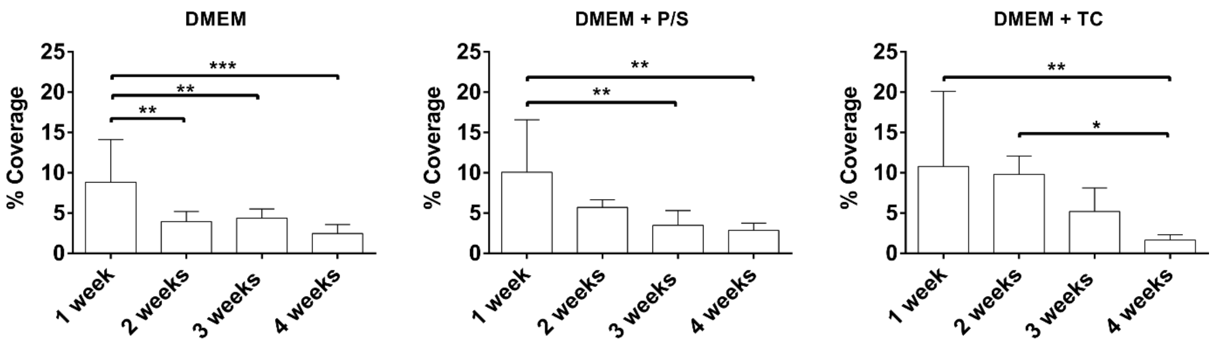

Supplementary Figure S1. Biofilm coverage after 1, 2, 3 and 4 weeks. *L. lactis*-FN biofilms were produced on PEA surfaces and cultured with DMEM, DMEM supplemented with 100 U/mL penicillin-streptomycin (P/S) and DMEM supplemented with 10 µg/mL tetracycline (TC). After selected time points, biofilms were washed and subjected to a viability analysis with BacLight, a commercial viability kit. Biofilms were imaged under an epifluorescence microscope and their coverage on the surface was analysed with Fiji / ImageJ. Data shows that there is a decrease in area coverage with time, and, since this decrease is mostly due to detachment of non-viable cells, this result explains the increase in the viability values found in the Figure 2.

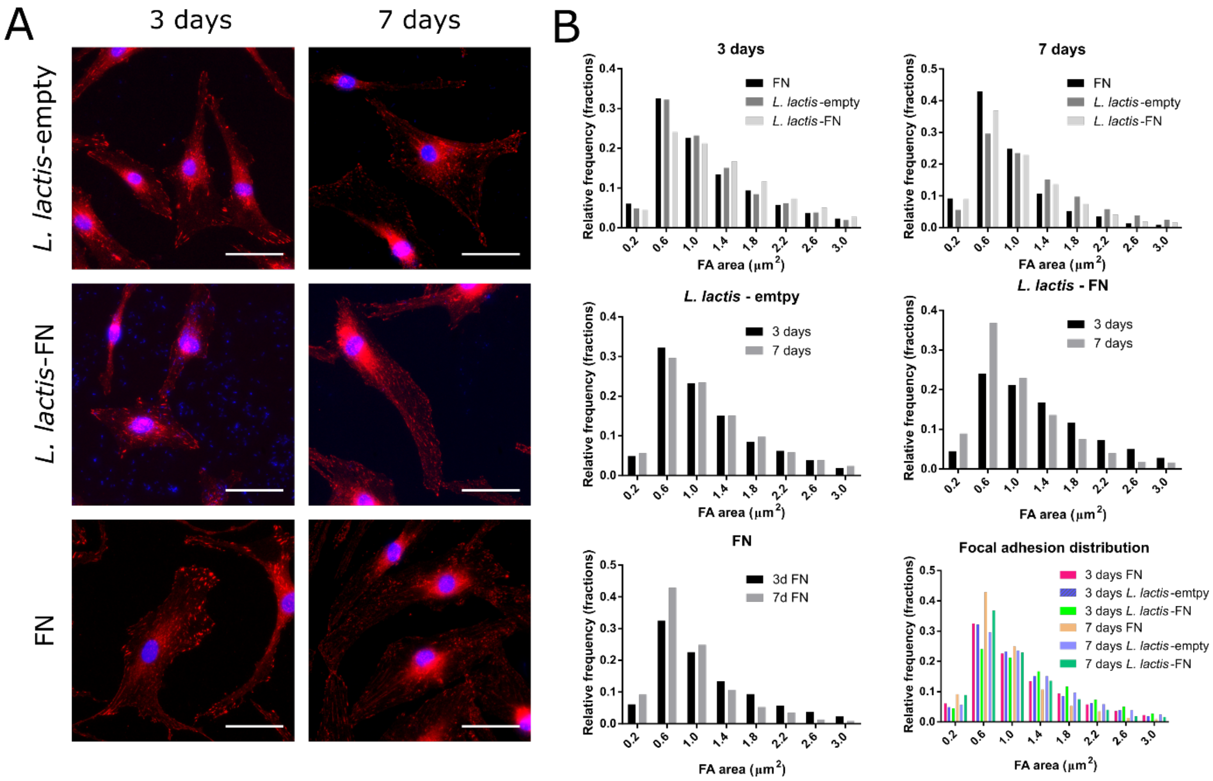

Supplementary Figure S2. Focal adhesion analysis was performed on hMSCs after 3 and 7 days of culture on *L. lactis*-empty, *L. lactis*-FN and FN-coated surfaces. hMSCs were cultured in DMEM supplemented with 10 µg/mL tetracycline and 1% FBS and after the selected time points, fixed and immunostained against vinculin (red), while nuclei were stained with DAPI (blue). A minimum of 12 individual, isolated cells per condition were analysed to check area and number of focal adhesion complexes. Results are detailed on the graphs. There are no differences in focal adhesion distribution between time points nor surfaces, probably due to the fact that the FBS used in the cell culture medium is helping in the initial adhesion steps, via the fibronectin, vitronectin and collagen present in the serum. After this initial adhesion, hMSCs start to secrete their own ECM proteins, leading to a very similar behaviour in all of the tested conditions. Scale bar size is 50 µm.

Supplementary videos. hMSCs were cultured on *L. lactis*-FN and *L. lactis*-empty biofilms at 5,000 cells/cm^2 and imaged in a phase-contrast time-lapse microscope (EvosFL Auto, Life Technologies). Images were captured at 10x every 15 minutes for a total of 18 hours, and compiled in a video. Video 1 corresponds to hMSCs cultured on the *L. lactis*-empty biofilm with 1% FBS to ensure long-term viability and 10 µg/mL TC to prevent medium acidification. Video 2 corresponds to hMSCs cultured on the *L. lactis*-FN biofilm in the same conditions. Video 1 shows that cells remain up to 2 hours in a round shape, indicative of initial absence of adhesive behaviour. After 2 hours, due to the presence of FBS in the medium, cells start to attach and spread, although their mobility on the biofilm is lower when compared to cells cultured on the *L. lactis*-FN biofilm. Video 2 shows that cells attach and spread in 45 minutes and keep migrating over the biofilm for 18 hours. This behaviour suggest that the exposed $FNIII_{7\text{-}10}$ fragment on the *L. lactis* cell wall helps hMSCs in their migration over the biofilm.
